# Free circulating versus extracellular vesicle-associated microRNA expression in canine T-cell lymphoma

**DOI:** 10.3389/fvets.2024.1461506

**Published:** 2024-08-29

**Authors:** Cecilia Capuano, Valentina Moccia, Antonella Molinari, Filippo Torrigiani, Livia Ferro, Serena Ferraresso, Federico Bonsembiante, Chiara Leo, Valentina Zappulli

**Affiliations:** ^1^Anicura Istituto Veterinario di Novara, Granozzo Monticello, Italy; ^2^Department of Comparative Biomedicine and Food Science, University of Padua, Legnaro, Italy; ^3^Department of Animal Medicine, Production and Health, University of Padua, Legnaro, Italy

**Keywords:** extracellular vesicles, miRNA, liquid biopsy, veterinary oncology, canine lymphoma

## Abstract

**Introduction:**

Canine lymphoma (cL) is one of the most frequent cancers in dogs. The T-cell lymphoma (TcL) is not the most common phenotype but presents an aggressive behavior. MicroRNAs (miRNAs), are small, single-stranded, non-coding RNA molecules which can circulate freely in blood or be associated with extracellular vesicles (EVs). The dysregulation of certain miRNAs has been identified in numerous types of human cancers and they have been largely investigated as possible tumors biomarkers in human medicine, while research in veterinary oncology is still scarce. The aim of this study was to compare the expression patterns of free circulating and EV-associated miRNAs in dogs with T-cell lymhoma (TcL) and healthy dogs.

**Methods:**

Eight dogs with TcL were selected as the lymphoma group (LG) and eight dogs were included as controls (Ctrl). Plasma samples were collected at the time of the diagnosis and EVs isolated with ultracentrifugation. miRNAs were extracted from both the circulating EVs and the plasma supernatant, obtaining EV-associated and free-miRNAs. Quantitative real-time PCR was performed to analyze the expression of 88 target miRNAs.

**Results:**

Ten and seven differentially expressed miRNAs between LG and Ctrl were detected in EV-associated and free-miRNAs, respectively. Among EV-associated and free-miRNAs, only has-miR-222-3p was overexpressed in both conditions.

**Discussion:**

All the differentially expressed miRNAs detected in this study, have been already described as dysregulated in other human or canine cancers. The EV-associated miRNAs, which appear to be more stable and better conserved than free-miRNAs, could be investigated in further larger studies to better assess their use as possible biomarkers for TcL.

## 1 Introduction

Canine lymphoma (cL) is among the most frequently diagnosed cancers in dogs and represents the most managed neoplasia in veterinary oncology (1). Most of the cases are B-cell lymphoma (70%), while the others can be T-cell lymphoma (TcL) or non-B/non-T cell lymphoma (1, 2). Generally, cL is routinely diagnosed by cytologic examination of affected tissues but other diagnostic investigations may be pursued for immunophenotyping, grading and clinical staging, mainly to predict the biological behavior (2). Multi-agent chemotherapy represents a gold standard treatment for cL and remission is often achieved, despite TcL can develop more commonly drug resistance (2).

MicroRNAs (miRNAs) are small non-coding RNA molecules of approximately 22 nucleotides in length that participate in post-transcriptional gene regulation. Having this role, they are involved in the regulation of different biological processes, including carcinogenesis being either oncogenes or tumor suppressors (3). Many studies have demonstrated that the expression of many oncogenic miRNAs is dysregulated in tumors, enhancing proliferative signaling, evasion from growth suppressors, cell death resistance, invasiveness, and angiogenesis (3). Many mechanisms are involved in this dysregulation and include amplification or deletion of miRNA genes, abnormal transcriptional control of miRNAs, dysregulated epigenetic changes, and defects in the miRNA biogenesis machinery (3).

Given that miRNAs exist not only in cells and tissues but also across various body fluids, coupled with their significant stability, they offer a vast resource of minimally invasive biomarkers (2). Moreover, miRNAs can circulate in the blood freely or associated with extracellular vesicles (EVs).

Extracellular vesicles (EVs) are heterogenous membrane-bound vesicles released by cells (4). EVs, which transport molecules like proteins, sugars, lipids, and nucleic acids, play a crucial role in cell-to-cell communication (5).

EVs are mainly divided into exosomes and microvesicles, according to their biogenesis (5, 6). However, other cell-derived particles can be categorized as EVs, including for example apoptotic bodies, prostasomes, and oncosomes. Considering the heterogeneity of EVs and the difficulties in recognizing their origin, they are usually also classified according to their size in small-EVs (<200 nm) or large-EVs (>200 nm) (5, 6).

Among many other functions, EVs play a role in promoting the carcinogenic process, inducing angiogenesis, tumor dissemination, immune escape, metastasis, and drug resistance (5, 7, 8). EVs are present in all body fluids (e.g., blood, saliva, urine, bronchoalveolar fluid, breast milk, semen) and being stable for long time and are easy to sample, being suitable for sequential collection (8). EV-associated RNAs have been reported as possible tumor biomarkers for cancer diagnosis or for monitoring cancer progression in many studies in human medicine (8). However, even though there have been some studies in veterinary medicine, knowledge remains limited regarding the expression of both free circulating and EV-associated miRNAs in dogs with lymphoma (2, 9, 10).

Considering the need of non-invasive biomarkers in veterinary oncology and the paucity of information regarding miRNA expression in canine T-cell lymphoma (TcL), the aim of this study was to identify expression patterns of specific circulating miRNAs in dogs with TcL, investigating both free circulating miRNAs and EV-associated miRNAs as possible diagnostic biomarkers.

## 2 Materials and methods

### 2.1 Case recruitment

This was a prospective double-arms study. Eight dogs were included as controls (Ctrl) and eight dogs diagnosed with TcL were selected according to eligible criteria for the lymphoma group (LG). All LG and some Ctrl samples were provided by Anicura Istituto Veterinario Novara (Novara, NO, Italy). The remaining Ctrl samples were provided by the Veterinary teaching hospital of the University of Padua (Legnaro, PD, Italy). All lymphoma patients were treated at the AniCura Istituto Veterinario Novara with chemotherapy protocols that required systematic hematology controls. Blood samples were obtained at the time of diagnosis (T0), during the clinical diagnostic routine procedures and leftovers were used for the study. Blood samples from Ctrl were obtained during elective surgery for sterilization and leftovers were kept for the study. Approval by an ethics committee was therefore not required for this study.

Inclusion criteria for the Ctrl included: no clinical, hematology or biochemistry abnormalities. Inclusion criteria for LG included a cytological or histological diagnosis of lymphoma, no previous treatments with chemotherapy or steroids, and absence of other comorbidities. The presence or suspicion of leukemic lymphoma was considered exclusion criteria. Informed consent was obtained from all owners.

The diagnosis, grading, and staging of lymphoma were performed according to the Kiel updated classification (Lennert K, Feller CA: Histologie des Lymphomes Malins Non Hodgkiniens Selon la Classification de Kiel Actualise´. Doin, Paris, France, 1991). For each case, clinical examination, complete blood count (CBC) and biochemistry profile, chest radiographs, abdominal ultrasound (US) and cytology of the lymph nodes enlarged at clinical presentation, liver (with one exception) and spleen were performed according to standard diagnostic procedures. To confirm the T-cell origin of lymphomas, the immunophenotyping was assessed by flow cytometry and the grading was determined by the size of cells (majority of small-sized cells or majority of medium- and large sized cells) and mitotic index (11). The cL phenotypes were determined by flow cytometry (CyFlow Space, Sysmex Europe GmBH, Norderstedt, Germany) and the data analyzed with the FlowMax software (Sysmex Europe GmBH, Norderstedt, Germany). For flow cytometry, lymph node samples were collected in Eppendorff containing 500 μL of autologous serum and analyzed within 24 h from collection. Erythrocyte lysis was performed using erythrocyte lysis buffer containing 8% ammonium chloride if deemed necessary due to hemodilution. The percentages of dead and live cells were assessed using propidium iodide (PI) to evaluate samples’ preservation before the staining with antibodies. A panel of antibodies that included CD45 and CD44 (pan-leukocyte), CD3 and CD5 (T lymphocytes), CD4 (T-helper lymphocytes), CD8 (T-cytotoxic lymphocytes), CD21 (B lymphocytes), CD34 (blast cells) was used. For each sample, 50 μL of cell suspension were added to the tubes containing different combinations of antibodies (Supplementary Table S1).

### 2.2 Isolation and characterization of extracellular vesicles from plasma and microRNA extraction

A total of 4 mL of venous blood were collected by each dog and transferred to an EDTA tube. Upon collection, each sample was centrifugated at 4°C at 2000 × *g* for 15 min to obtain the plasma fraction. The plasma was stored at −80°C until isolation of EVs.

EVs were isolated from plasma of Ctrl and LG dogs with ultracentrifugation (UC). Each sample was first centrifuged at 4°C (2000 × *g* for 10 min) to remove large debris. The supernatant was then transferred to a new tube and centrifuged twice at 3850 × *g*. The final supernatant (about 1 mL) was diluted in phosphate-buffered saline (PBS) 0,2 μm double- filtered (dfPBS) to reach a final volume of 4 mL, transferred to an ultracentrifuge tube (Ultra-Clear Open top, Beckman Coulter, Brea, CA, USA) and ultracentrifuged in a swinging bucket rotor (SW55ti, Beckman Coulter, Brea, CA, USA) at 100.000 × *g* for 90 min at 4°C (Beckam Coulter Optima L-90K, Beckman Coulter, Brea, CA, USA).

After UC, the obtained pellets containing EVs (EV-pellet) were lysed and nucleic acids extracted using miRNAeasy^®^ Micro Kit (Qiagen, Hilden, Germany) according to the manufacturer’s instructions to finally obtain EV-associated miRNAs. To extract free circulating miRNAs (free-miRNAs), miRNaeasy Serum/Plasma Advanced Kit (Qiagen, Hilden, Germany) was used according to the manufacturer’s instructions on UC-supernatant.

Because of the low volume of plasma samples, two more Ctrl samples, not included in the following miRNA analysis, were used as techinical controls to assess EV-presence after UC. After performing EV-isolation as described above, one of these two samples was used to perform Nanoparticle Tracking Analysis (NTA) and one for Western Blotting (WB).

### 2.3 Nanoparticle tracking analysis

NTA was performed to assess the size distribution and concentration of EVs. After UC, the EV-pellet was resuspended in 600 μL of dfPBS and then diluted 1:100 in the same buffer. NTA was performed with NanoSight NS300 (Malvern Panalytical, Malvern, United Kingdom) and 3 videos of 60 s each were recorded and analyzed using software 3.4, with camera level set at 14 and detection threshold at 5. Values considered reliable were those included in the following ranges: particles per frame 20 to 120; particle concentration: 10^6^–10^9^/mL; total particles to valid particles ratio ≥ 1/5.

### 2.4 Western blotting

WB was performed to verify the presence of EV-markers in the EV-enriched pellet.

After UC, proteins from EV-enriched pellet were extracted using 20 μL of RIPA buffer (Thermo Fisher Scientific, Waltham, MA, USA) supplemented with proteinase inhibitor. First, protein concentration was quantified by Pierce BCA protein Assay Kit buffer (Thermo Fisher Scientific, Waltham, MA, USA), according to manufacturer’s instructions. 20 μg of proteins were denatured for 10 min at 70°C, resolved by electrophoresis gel using NuPAGE 4-12% Bis Tris gel (Thermo Fisher Scientific, Waltham, MA, USA) and transferred on to a nitrocellulose membrane. Non-specific binding sites were blocked by a 90 min incubation at room temperature with a TBS-T solution (Tris-buffered saline with 0.05% of Tween-20) supplemented with 5% of skimmed milk powder.

Blots were incubated overnight in TBS-T supplemented with 1% of skimmed milk with primary anti-human antibodies against TSG101 (cytosolic protein, dilution 1:1000, GTX70255, GeneTex, Irvine, CA, USA) and integrin-beta (a membrane protein, dilution 1:5000, GTX128839, GeneTex, Irvine, CA, USA) at 4°C, a cytosolic and a membrane EV-markers, respectively (12). After overnight incubation, membranes were incubated at room temperature for 1 h with a peroxidase-conjugated secondary antibody (dilution 1:3000, anti-Rabbit #32260 or anti-Mouse #32230, Thermo Fisher Scientific, Waltham, MA, USA).

Finally, bands resulting from antigen–antibody binding were visualized using a chemiluminescent detection kit (SuperSignal West Pico PLUS Chemiluminescent Substrate, Thermo Fisher Scientific, Waltham, MA, USA) with the iBright instrument (Thermo Fisher Scientific, Waltham, MA, USA).

### 2.5 Reverse transcription and quantitative real-time PCR

The extracted RNA from each sample (8 LG subjects and 8 ctrl subjects) was reverse transcribed to complementary DNA (cDNA), using the miRCURY LNA RT Kit (Qiagen, Hilden, Germany) according to the manufacturer’s instructions. To each RNA, 0.5 μL of spike-in UniSp6 RNA was added as control for monitoring successful reverse transcription and to be used as inter-plate calibrator on qPCR. The obtained solution was incubated at 42°C for 60 min and then at 95°C for 5 min.

Finally, qPCR was performed immediately, or cDNA samples were stocked at −20°C for up to 5 weeks.

Quantitative RT-PCR was performed for each sample using a 96-well plate miRCURY LNA miRNA Focus PCR Panel (Qiagen, YAFD-201Z, Qiagen, Hilden, Germany), specifically designed by the manufacturer company for the diagnosis of canine lymphoma, and miRCURY LNA SYBR^®^ Green PCR Kit (Qiagen, Hilden, Germany), according to manufacturer’s instructions. These plates are designed to target 84 different miRNAs and 4 reference genes (U6 snRNA, 5S rRNA, RNU5G, RNU1A1) while the remaining wells are for positive controls and interplate calibrators (i.e., IPC UniSp3) (Supplementary Table S2).

The reaction mix was prepared by adding 495 μL of Nuclease-free water, 510 μL of 2x miRCURY SYBR^®^ Green PCR Master Mix, 5.1 μL of ROX and 10 μL of cDNA. For each target/assay the qPCR reaction was carried out in a final volume of 10 μL. The amplification protocol consisted of an initial step of 2 min at 95°C, followed by 40 cycles of 10 s at 95°C and 60 s at 56°C. All experiments were carried out in a Thermo ABI 7500 (Applied Biosystems, Waltham, MA, USA).

### 2.6 Statistical analysis

The obtained cycle threshold values (Ct) were analyzed using the GeneGlobe miRNA PCR Array Data Analysis.^1^ To verify the efficiency of reverse transcription and qPCR reactions, the spike inscel-miR-39-3p and Unisp6 were used as internal amplification controls. A first control based on Ct value was applied on both target and reference miRNAs before normalization. Ct values above 36 were assigned the undetermined status, as the transcript is considered “undetectable.” miRNAs with Ct between 33 and 36 are considered “detectable but not quantifiable,” and miRNAs with Ct below 33 are considered “quantifiable” and thus usable for subsequent statistical analysis. A first inter-plate normalization was performed based on the IPC UniSp3. The software calculated the calibrator factor (*CF*), obtained from the difference between the Ct of the IPC of each sample (IPC plate) and the mean of the IPC plate values of all samples (IPC overall). For each sample, the Ct value of all genes was corrected (CtC) based on the *CF* value. The CtC values of each miRNA were then normalized based on the NormFinder method. Relative quantification was determined using the Delta\Delta Ct method. Fold change (FC) was calculated using 2^-(Delta\DeltaCT) to assess for differences in miRNA expression between the control and lymphoma group: FC greater than 1 were indicative of overexpression, while FC less than 1 represented under expression.

To compare the difference between the mean expression of each miRNA between the two groups, the Student’s *t*-test was performed and FC greater than 1.5 and *p* values (p) less than 0.05 were considered statistically significant.

## 3 Results

### 3.1 Patient characteristics

Sixteen dogs were enrolled in the study, eight dogs in the LG and 8 dogs in the Ctrl. The LG included four females and four males with a median age of 7.75 years (4–12) and belonging to various breeds (mixed, Boxer, Labrador retriever, Weimaraner, Bernese Mountain dog). The most common form of lymphoma was the multicentric one (five dogs), with one patient having nasal involvement as well. The other lymphoma presentations were mediastinal (one dog), hepatosplenic (one dog), cardiac (one dog) and in the jejunal lymph node (one dog) (Table 1).

**Table 1:** Characteristics of dogs in the lymphoma group.

ID	Gender	Age	Breed	Grade	Stage	Substage	Localization	Hypercalcemia
1	M	12	LR	HG	3	b	Multicentric	No
2	M	8	B	HG	5	b	Mediastinal	Yes
3	F	5	W	HG	4	b	Multicentric	No
4	F	4	M	HG	ND	b	Digiunal LN	Yes
5	M	6	B	HG	5	b	Hepatosplenic and cardiac	No
6	M	11	M	LG	5	a	Nasal and multicentric	No
7	F	6	BMD	HG	4	ND	Multicentric	No
8	F	10	M	HG	4	b	Multicentric	No

ID, assigned identity number; M, male; F, female; LR, Labrador Retriever; B, Boxer; W, Weimaraner; M, Mixed breed; BMD, Bernese Mountain dog; HG, high grade; LG, low grade; ND, not determined.

The cytological and flow-cytometric evaluation confirmed seven dogs (87.5%) presenting high-grade lymphoma, and one dog with low-grade lymphoma (ID 6). One dog had stage 3 lymphoma, three dogs had stage 4, and three dogs presented a stage 5 lymphoma. Six dogs (75%) were classified with substage b (Table 1).

### 3.2 Extracellular vesicle characterization

**Figure 1:**
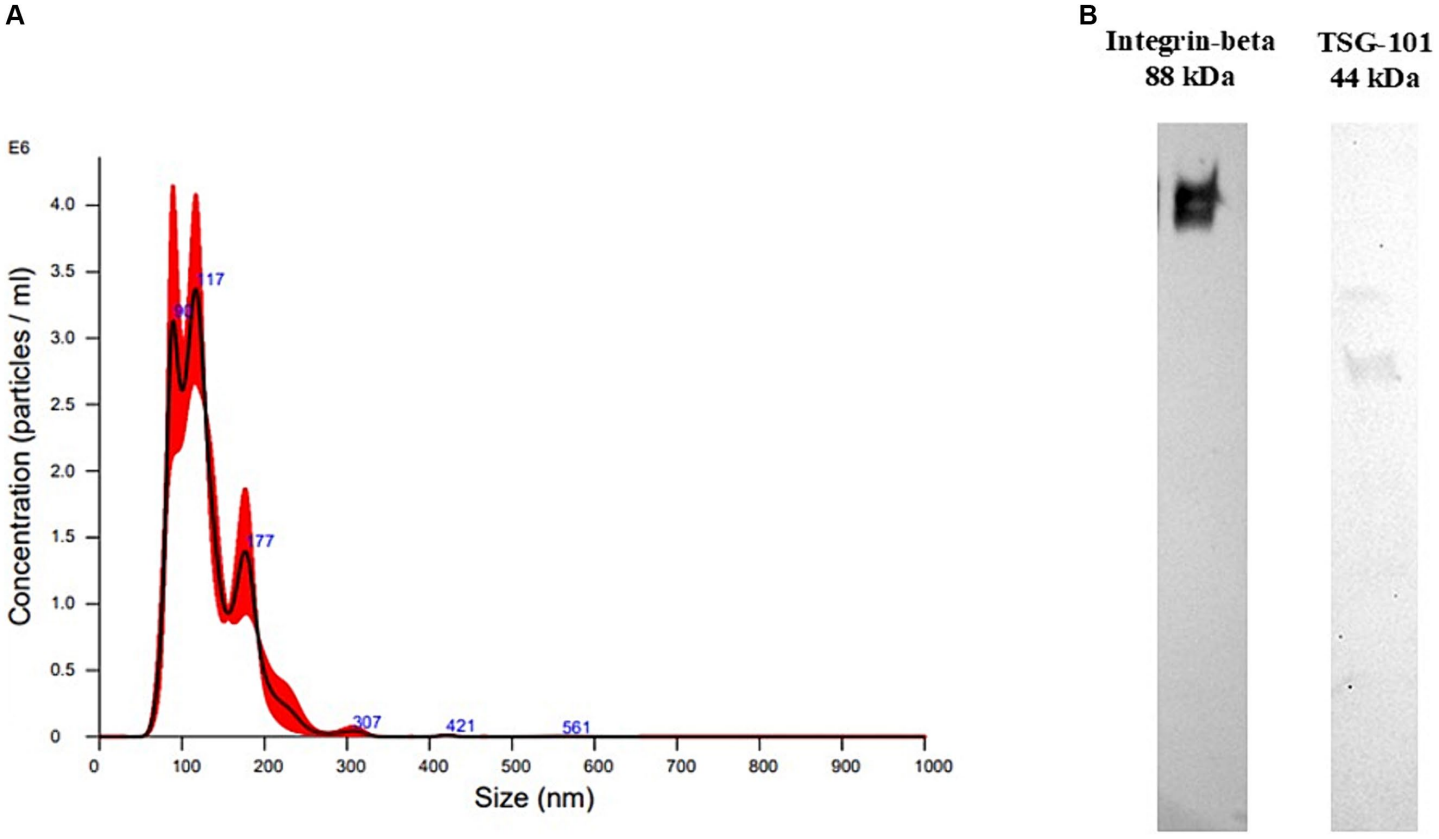
Extracellular vesicle characterization results. **(A)** Size distribution recorded at nanoparticle tracking analysis of plasma-derived extracellular vesicles isolated with ultracentrifugation. **(B)** Western Blotting performed on plasma-derived extracellular vesicles (EVs) isolated with ultracentrifugation. The sample was positive to TSG101, a cytosolic marker of EVs, and to integrin-beta, a membrane marker.

To confirm EV-size and concentration, NTA was performed on one EV-enriched pellet after UC (Figure 1A).

The particle size distribution showed ranges within those of EVs, mainly between 80 and 600 nm, with mean and mode having values of 130.4 +/− 3.1 and 111.3 +/− 12.3 nm, respectively, meaning that mainly small-EVs were isolated. Particle concentration was 2.53*10^12^ +/− 2.54*10^11^ particles/mL.

WB was performed on one sample to confirm the presence of EV markers in the EV-enriched pellet. The EV-enriched pellet resulted positive to two typical markers of EVs, TSG101, a cytosolic protein, and integrin-beta, a membrane protein (Figure 1B).

### 3.3 Quantification of extracellular vesicle-associated and free-circulating miRNAs

For each dog included into the study miRNA analyses were carried out both on EV-associated (EVs-LG and EVs-Ctrl) and free-miRNA (Free-miRNA-LG and Free-miRNA-Ctrl) samples.

The free-miRNA sample ID 6 (6-Free-miRNA-LG, Table 1) had CtC values for all target miRNAs much higher than all other samples, with a consequent low percentage of quantifiable miRNAs (<30%). Considering this insufficient percentage of quantifiable miRNAs, this sample was not included in the statistical analyses of the study. For all the other samples, the analysis of the amplification profiles identified a variable percentage of undetectable miRNAs, generally higher for free-miRNAs (missing data ranging from 7.29 to 20.83% for EV-associated miRNAs and from 12.50 to 46.88% for free-miRNAs) (Table 2). Among the EV-associated miRNAs and the free miRNAs, 5 miRNAs with the best stability score, were selected by the software as reference miRNAs for each of the two miRNA-sources (Supplementary Table S3). The coefficient of variation CV%, of the 5 reference miRNAs was calculated as the ratio of the standard deviation of each miRNA to its mean CtC among all samples, multiplied by 100. An optimal CV value is considered having value lower than 15%. In this study the calculated CV% for the reference miRNAs showed a value between 3 and 7% (Supplementary Table S3).

**Table 2:** Percentage (%) of missing data at the analysis of the amplification profile for each sample.

Sample	% of missing data
1_EVs_LG	13.54%
2_EVs_LG	11.46%
3_EVs_LG	11.46%
4_EVs_LG	13.54%
5_EVs_LG	11.46%
6_EVs_LG	15.63%
7_EVs_LG	7.29%
8_EVs_LG	17.71%
9_EVs_Ctrl	12.50%
10_EVs_Ctrl	18.75%
11_EVs_Ctrl	20.83%
12_EVs_Ctrl	14.58%
13_EVs_Ctrl	18.75%
14_EVs_Ctrl	14.58%
15_EVs_Ctrl	17.71%
16_EVs_Ctrl	15.63%
1_Free_miRNA_LG	33.33%
2_ Free_miRNA _ LG	34.38%
3_ Free_miRNA _ LG	12.50%
4_ Free_miRNA _ LG	29.17%
5_ Free_miRNA _ LG	41.67%
7_ Free_miRNA _ LG	34.38%
8_ Free_miRNA _ LG	22.92%
9_ Free_miRNA _Ctrl	27.08%
10_ Free_miRNA _Ctrl	46.88%
11_ Free_miRNA _Ctrl	42.71%
12_ Free_miRNA _Ctrl	37.50%
13_ Free_miRNA _Ctrl	27.08%
14_ Free_miRNA _Ctrl	43.75%
15_ Free_miRNA _Ctrl	42.71%
16_ Free_miRNA _Ctrl	40.62%

Each sample is described by the ID number followed by the miRNA source and the group of patients. EVs, plasma derived extracellular vesicles; free miRNAs, plasma free-circulating miRNAs; LG, canine T-cell lymphoma group; Ctrl, control group.

### 3.4 Differential miRNA expression between the two groups

A total of 10 EV-associated miRNAs were found to be differentially expressed in LG compared to Ctrl. One additional miRNA (miR-103a-3p) had a *p*-value close to significance (*p*-value = 0.0506) (Table 3 and Supplementary Figure S1).

**Table 3:** Extracellular vesicle-associated miRNAs significantly differentially expressed between the lymphoma and the control group.

MiRNA	Fold change	*p*-value
*hsa-miR-103a-3p*	0.67	0.009344
*cfa-miR-191*	1.71	0.000227
*hsa-miR-192-5p*	1.99	0.046515
*hsa-miR-222-3p*	2.88	0.002108
*rno-miR-223-3p*	2.18	0.034584
*bta-miR-27a-3p*	1.73	0.047376
*hsa-miR-30b-5p*	1.89	0.000372
*cfa-miR-30d*	2.05	0.032016
*hsa-miR-130a-3p*	3.35	0.050624
*hsa-miR-378a-3p*	2.92	0.022901

A total of seven free miRNAs were found to be differentially expressed in LG compared to Ctrl (Table 4 and Supplementary Figure S1).

**Table 4:** Free circulating miRNAs significantly differentially expressed between the lymphoma and the control group.

MiRNA ID	Fold change	*p*-value
*hsa-miR-106b-5p*	1.60	0.017385
*hsa-miR-146a-5p*	3.62	0.038298
*gga-miR-18a-5p*	2.35	0.013136
*hsa-miR-21-5p*	3.23	0.002350
*hsa-miR-222-3p*	2.50	0.030518
*hsa-miR-93-5p*	1.62	0.021672
*hsa-let-7b-5p*	−1.98	0.005933

**Figure 2:**
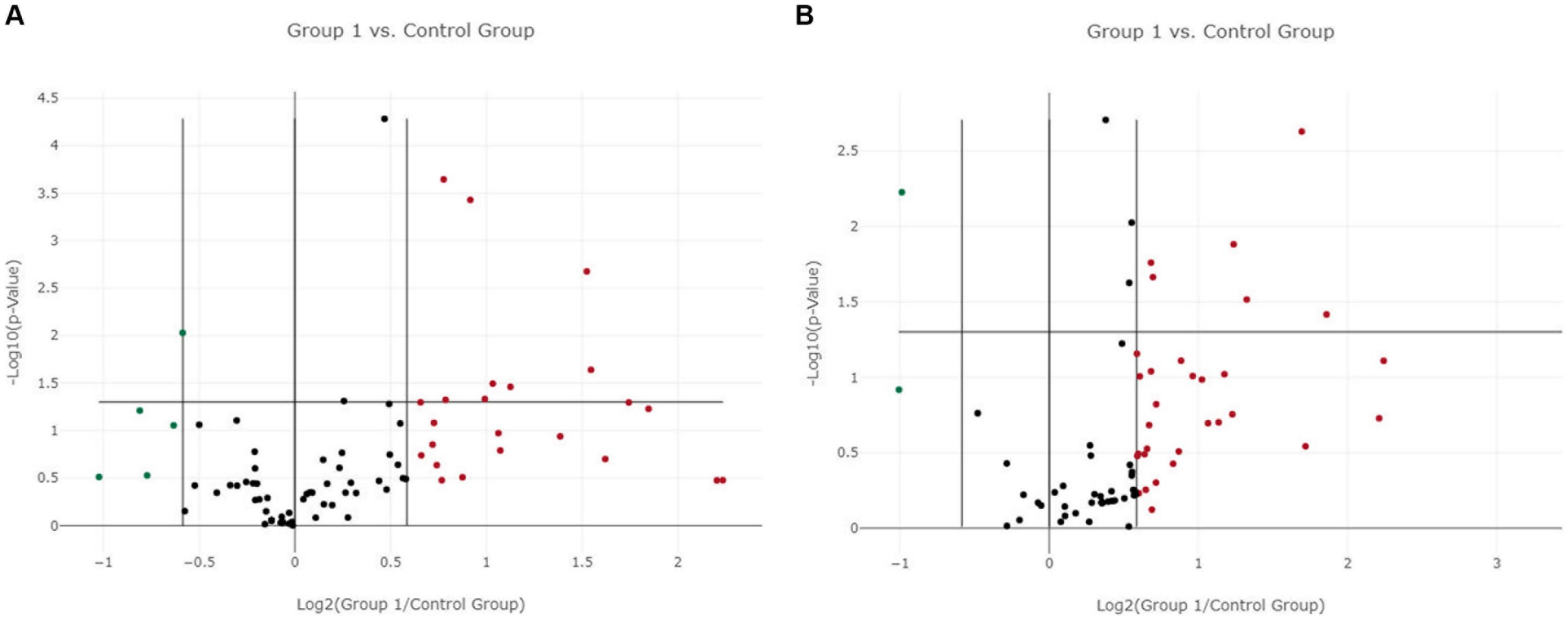
Volcano plot of differentially expressed genes between the lymphoma and the control group. **(A)** Extracellular vesicle-associated miRNAs. **(B)** Free circulating miRNAs. Group 1 = lymphoma group.

In LG, only one miRNA (miR-222-3p) was found as overexpressed both in EV-associated and free-miRNAs (Tables 3, 4). As shown in the volcano plot, among the differentially expressed EV-associated miRNAs in LG, 8 were statistically significantly overexpressed, one was significantly under-expressed, and one was overexpressed but marginally significant (Figure 2A).

Among the differentially expressed free miRNAs, 6 miRNAs were statistically significantly overexpressed, and one was significantly under-expressed in the LG (Figure 2B).

**Figure 3:**
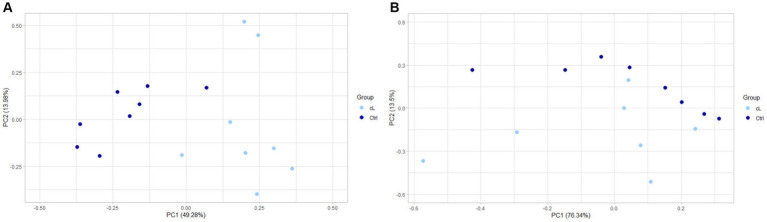
PCA of differentially expressed genes between the lymphoma and the control group. **(A)** Extracellular vesicle-associated miRNAs. **(B)** Free circulating miRNAs. cL, lymphoma group; Ctrl, control group.

Finally, a principal component analysis (PCA) on differentially expressed genes in EV-associated miRNA samples, showed a separation between LG and Ctrl (Figure 3A). PCA performed on differentially expressed genes of the free-miRNA samples did not show instead any separation between LG and Ctrl (Figure 3B).

## 4 Discussion

Canine TcL continues to pose significant challenges for veterinary oncologists, with a particularly rapid and aggressive behavior and no curative approaches developed in the past 25 years despite numerous attempts (1, 13). Similar to other pet tumors, early detection remains elusive, and the focus on biomarker research is seen as a beacon of hope for identifying lymphomas at an early stage, or to provide prognostic value. In this context, *in vivo* studies are crucial, as *in vitro* research on cancer cells fails to identify the early stages of the disease and does not accurately replicate the complexity of *in vivo* conditions (14, 15).

MiRNAs have gained popularity in veterinary and human medicine as tumor minimally invasive biomarkers, given their stable presence not only in cells and tissues but also across various body fluids (2, 3). This preliminary study aimed to identify the expression patterns of circulating miRNAs in a small group of canine T-cell lymphoma dogs, and compare it to healthy dogs, investigating the expression of both free circulating miRNAs and EV-associated miRNAs.

EV-characterization performed on two technical controls, showed that EV-isolation with UC allowed the purification of particles that were similar in terms of concentration and size distribution to those of other studies in which mainly small-EVs were isolated using UC from canine plasma (16, 17). Moreover, the isolated particles were positive to two typical EV-markers at WB, a cytosolic (TSG101) and a membrane marker (integrin-beta) (6).

After EV characterization, we conducted qPCR and data analysis on both EV-associated and free circulating miRNAs. As general considerations, more undetermined Ct values were detected in the free circulating miRNA samples compared to the EV-associated ones, meaning that less miRNAs were overall detected in the free-miRNA samples. Furthermore, there was a marginally higher number of differentially expressed miRNAs in EV-associated compared to free-miRNAs when comparing LG and Ctr (9 vs. 7). Among the differentially expressed miRNAs, only one was in common between EV-associated and free-miRNA samples (miR-222-3p). This lack of correspondence between the differentially expressed miRNAs that were either EV-associated or free in the plasma might be due to the fact that EV-associated miRNAs constitute only a small portion of the total plasma miRNAs and their expression can differ from the free circulating ones (18, 19). Moreover, EV-associated miRNAs are more stable and better shielded from RNAse activity, which could account for the higher amount of determined Ct values observed in EV-associated miRNAs in our study (20, 21). Consistently with our results, a recent review underscored that among numerous studies examining miRNA expression in human prostate cancer patients, either as total circulating miRNAs (including both EV-associated and free-miRNAs) or EV-associated miRNAs, only miR-21 was identified as overexpressed in both miRNA sources across different patient groups (22). Since usually studies analyze only EV-associated miRNAs or the total circulating miRNAs, to our knowledge, our work is the only one comparing the EV-associated miRNAs and the free-circulating miRNAs extracted from the EV-depleted plasma, in the same group of patients (22).

While literature on cancer both circulating and EV-associated miRNAs is abundant in human medicine, fewer studies have been performed in veterinary medicine (2, 9, 10, 23–25).

Garnica and coauthors investigated the relationship between serum EV-associated miRNAs and the response to therapy in dogs with multicentric lymphoma (MCL). Among the investigated miRNAs, the authors found mir-222 being more expressed in the group that achieved complete response, and mir-93 being overexpressed in the group showing progressive disease (9). In our study, miR-222 was found to be overexpressed in both EV-associated and free-miRNAs in the LG. On the other hand, miR-93 showed overexpression in free-miRNAs, but not in the EV-associated ones. The discrepancy in EV-associated miRNA profiles between our study and that of Garnica and colleagues might stem from several factors: (i) their specific focus on MCL involving both B and T cells, (ii) their exclusive examination of small-EVs, and (iii) their analysis of serum-derived EVs as opposed to plasma-EVs, matrixes which have been shown to possess different EV profiles (9).

Circulating miRNAs in lymphoma bearing dogs were also examined in another study, which focused on the analysis of total circulating serum miRNAs, encompassing but not differentiating between free- and EV-associated miRNAs (10). In this study, the only miRNA that was differentially expressed and consistent with our findings was miR-let-7b, downregulated in cL cases in both studies and differentially expressed as free-miRNA in our analysis. Once again, different results could be attributed again to different case selection criteria (exclusively TcL versus various types of lymphomas) and the distinction between analyzing EV-associated miRNAs and total circulating serum miRNAs (10).

Lastly, also miR-18a, which was overexpressed among the free-miRNAs in our study, has been reported as overexpressed in dogs with cancer, specifically in the serum of dogs with mammary carcinoma (26).

According to the literature, all miRNAs identified as differentially expressed in both free- and EV-associated samples in this work, have been previously noted for their overexpression or downregulation in human tumors and appear to play an important role in both tumor oncogenesis and oncosuppression (27–38). Indeed, some of the miRNAs that we found differentially expressed in TcL have been reported as markers in human lymphoma. miR-21 and miR191 have been found to be overexpressed in the serum of patients with advanced stages of diffuse large B-cell in lymphoma (DLBCL), and miR21 has also been reported being overexpressed in plasma-EVs of patients with Hodgkin lymphoma (39, 40). miR-30d, that was found overexpressed in EV-associated miRNAs, was at contrary downregulated among the total circulating serum miRNAs of human patients with chronic lymphocytic leukemia (41). Interestingly, miR-let-7b and miR-18a, previously identified as dysregulated in canine lymphoma and canine mammary carcinoma respectively, have also been observed as dysregulated in the total circulating serum miRNAs of human patients suffering from DLBCL. Notably, miR-let-7b has been linked with more advanced stages of this disease (10, 25, 42).

The other miRNAs found as differentially expressed in our study, which have not been reported in canine malignancies or human lymphoma so far, have been investigated in other types of human cancers, and some of them have been detected as associated to circulating EVs. miR-146 and miR-223, that we found as overexpressed in free- and EV-associated miRNAs respectively, were underexpressed in serum-EVs of human patients with laryngeal squamous cell carcinoma (43). miR-223 has been found overexpressed and downregulated in patients with colorectal and epithelial ovarian cancer, respectively, when EV-associated (44, 45). In colorectal cancer patients, also miR-192 and miR-27a have been found to be differentially expressed when EV-associated and miR-30b, overexpressed among EV-associated miRNAs in our study, was found underexpressed in plasma EVs of women with breast cancer recurrence (44, 46, 47).

Lastly, miR-103a and miR-130a, which were, respectively, downregulated and overexpressed when EV-associated in our study, have been found overexpressed in the total circulating serum miRNAs of human patients with lung cancer and urinary bladder cancer, respectively (48, 49). miR-106, that we have found overexpressed among free-miRNAs, has been considered a potential circulating diagnostic biomarker for hepatocellular carcinoma and a prognostic biomarker for gastric cancer (50).

While the findings of this study align to some extent with prior research in human and canine oncology, suggesting a subset of miRNAs as potential early diagnostic biomarkers for TcL, we have to acknowledge some limitations of this study. A limited number of patients was included due to difficulties in obtaining blood samples from a homogenous subset of subjects. Additionally, to exclude patients that had undergone different treatment protocols, samples were only gathered at the time of diagnosis. Consequently, to achieve more substantial results, further investigations should involve a larger, uniform group of individuals including additional samples collected at different timing post-diagnosis. These investigations could focus on the analysis of the herein found differentially expressed miRNAs, evaluating their efficacy not only as diagnostic, but also as prognostic and predictive biomarkers. This would involve adding follow-up data from larger sample groups. Moreover, although UC has always been considered the gold standard method for EV isolation and is a very well-established technique that we an excellent margin of certainty allows isolation of EVs, we could analyze EV concentration, size and characterization only on two samples used as procedural controls. Further, there are other techniques that, according to the literature, allow the collection of purer samples being less operator-dependent (6, 51, 52). Therefore, future studies could also consider using and comparing different EV-isolation techniques possibly applying them to the whole subset of analyzed samples.

## 5 Conclusion

This preliminary prospective double-arms study compares for the first time the expression of EV-associated and free-circulating miRNAs in the plasma of dogs with TcL and healthy control dogs. Ten EV-associated and seven free circulating miRNAs were found differentially expressed between LG and Ctrl, with hsa-miR-222-3p being overexpressed in both EV-associated and free circulating miRNAs. Several miRNAs identified as differentially expressed in this study have previously been recognized as potential markers in TcL, making them promising candidates for more in-depth analysis. The choice of using EV-associated miRNAs as biomarkers over free circulating miRNAs would have several advantages, as EV-content is stable and protected from degradation by circulating RNAse. The miRNAs found in this study could be used in further studies with a larger number of patients and additional follow-up and therapeutic information to investigate their possible use also as prognostic and predictive biomarkers. These preliminary results add new insights into EV-associated miRNAs within the field of veterinary medicine, enhancing our understanding of their expression in TcL. Our expectations are to contribute to the development of quicker, more effective, and innovative diagnostic and therapeutic tools.

## Data Availability

The original contributions presented in the study are included in the article/Supplementary material, further inquiries can be directed to the corresponding author.

## Footnotes

^1^www.geneglobe.qiagen.com/analyze
