# Supplementary figures and images for: Free circulating versus extracellular vesicle-associated microRNA expression in canine T-cell lymphoma
Source: Front Vet Sci. 2024 Aug 29;11:1461506. doi: 10.3389/fvets.2024.1461506 (PMC11390581; doi:10.3389/fvets.2024.1461506)

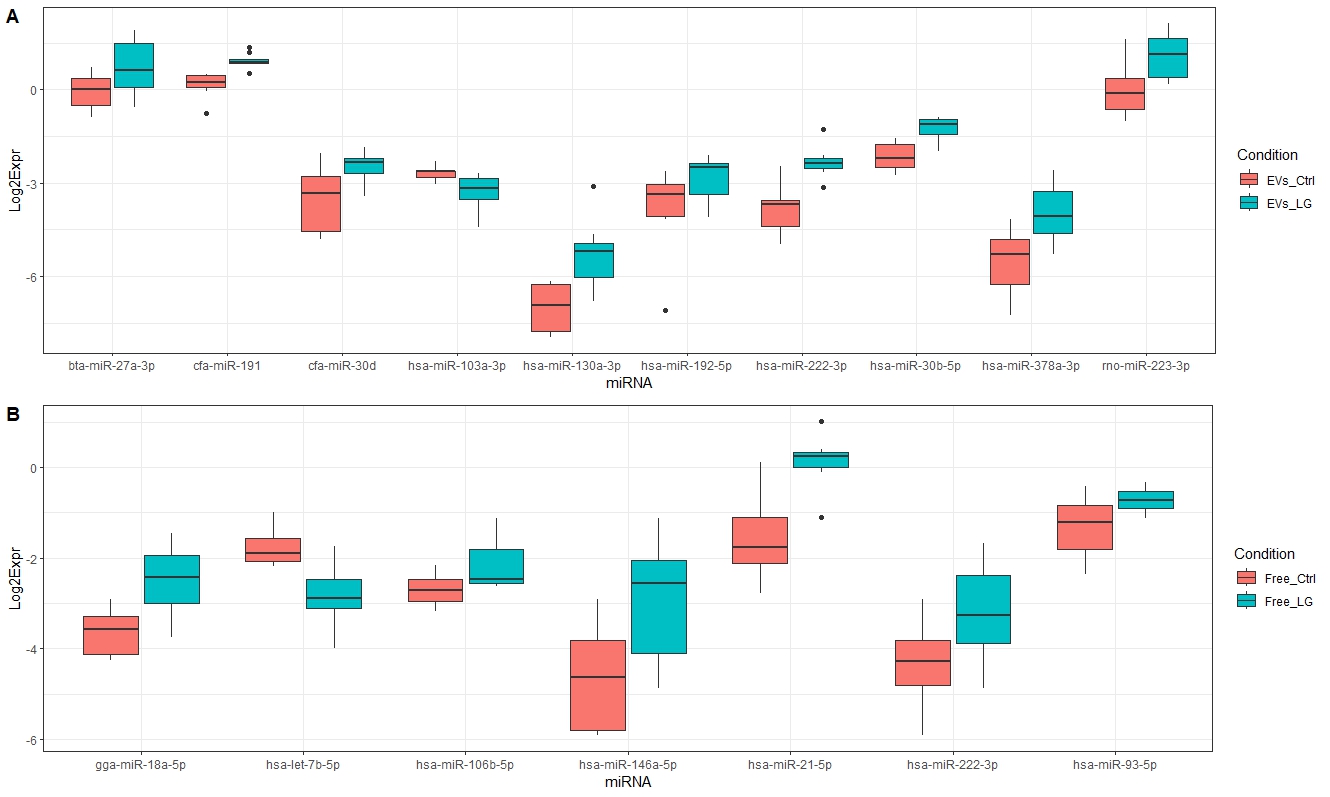

Supplement: Supplementary file 1 [file Image_1.jpeg]
