# Supplementary material for: Free circulating versus extracellular vesicle-associated microRNA expression in canine T-cell lymphoma
Source: Front Vet Sci. 2024 Aug 29;11:1461506. doi: 10.3389/fvets.2024.1461506 (PMC11390581; doi:10.3389/fvets.2024.1461506)
Supplement: Supplementary file 2 [file Table_1.docx]

**Table S1:** antibodies combinations used to determine canine lymphoma (cL) phenotypes. In brackets the dilution used. Abbreviations. FITC: fluorescein isothiocyanate, PE: phycoerythrin, APC: allophycocyanin, CD: cluster of differentiation.

| Tubes | FITC-conjugated | clone | PE-conjugated | clone | APC-conjugated | clone |
| --- | --- | --- | --- | --- | --- | --- |
| 1 | CD45 (1:200) | YKIX716.13 | CD5 (1:150) | YKIX322.3 | CD21 (1:200) | MCA1781A647 |
| 2 | CD4 (1:150) | YKIX302.9 | CD5 (1:150) | YKIX322.3 | CD8 (3µL undiluted) | YCATE55.9 |
| 3 | CD3 (1:10) | CA17.2A12 | CD4 (1:25) | YKIX302.9 | CD8 (3µL undiluted) | YCATE55.9 |
| 4 |  |  | CD34 (1:500) | 1H6 | CD44 (1:100) | IM7 |
