# Supplementary material for: Free circulating versus extracellular vesicle-associated microRNA expression in canine T-cell lymphoma
Source: Front Vet Sci. 2024 Aug 29;11:1461506. doi: 10.3389/fvets.2024.1461506 (PMC11390581; doi:10.3389/fvets.2024.1461506)
Supplement: Supplementary file 4 [file Table_3.docx]

**Table S2.** Reference miRNAs calculated for the extracellular vesicle-associated miRNAs (EVs) and free circulating miRNAs (free-miRNAs) groups and corresponding mean cycle threshold (Ct), standard deviation and coefficient of variation (CV%).

| **Sample** | **ID MiRNA** | **Mean Ct** | **Standard deviation** | **Coefficient of variation (%)** |
| --- | --- | --- | --- | --- |
| *EVs* | *has-let-7g-5p* | 26.13 | 0,97 | 3.72 |
|  | *has-miR-16-5p* | 22.02 | 0,91 | 4.15 |
|  | *cfa-miR-106a* | 25.40 | 0,91 | 3.59 |
|  | *has-miR-103a-3p* | 28.24 | 0,95 | 3.38 |
|  | *has-miR-20a-5p* | 24.50 | 0,86 | 3.52 |
| *Free-miRNAs* | *hsa-miR-92a-3p* | 30.22 | 1.46 | 4.8 |
|  | *hsa-miR-19a-3p* | 29.56 | 1.68 | 5.7 |
|  | *cfa-miR-106a* | 26.78 | 1.52 | 5.7 |
|  | *hsa-let-7b-5p* | 22.80 | 1.52 | 6.7 |
|  | *hsa-miR-451a* | 27.44 | 1.56 | 5.7 |
