# Supplementary material for: Free circulating versus extracellular vesicle-associated microRNA expression in canine T-cell lymphoma
Source: Front Vet Sci. 2024 Aug 29;11:1461506. doi: 10.3389/fvets.2024.1461506 (PMC11390581; doi:10.3389/fvets.2024.1461506)
Supplement: Supplementary file 5 [file Table_4.docx]

Supplementary Material

# Supplementary Figures and Tables

# Supplementary Table S2

**Table S2.** Reference miRNAs calculated for the extracellular vesicle-associated miRNAs (EVs) and free circulating miRNAs (free-miRNAs) groups and corresponding mean cycle threshold (Ct), standard deviation and coefficient of variation (CV%).

**1.2 Supplementary Figure S1**

**Figure S1. Box plot of** differentially expressed genes between the lymphoma and the control group. (A) Extracellular vesicle-associated miRNAs; (B) free circulating miRNAs. EVs_Ctrl = extracellular vesicle-associated miRNAs in control group; EVs_LG = extracellular vesicle-associated miRNAs in lymphoma group; Free_Ctrl = free circulating miRNAs in control group; Free_LG = free circulating miRNAs in lymphoma group.
